# Herpes Zoster with disseminated lesions. What is it?

**Published:** 2013-03-25

**Authors:** S Gupta, S Gupta, M Thomas, A Mahendra, N Jindal, G Bhaskar, M Aggarwal

**Affiliations:** * Department of Dermatology and STI, MM Institute of Medical Sciences and Research, Mullana Ambala- India; ** Department Of Medicine, MM Institute of Medical Sciences and Research Mullana Ambala- India; *** Department of Dermatology, Bangalore India; **** Aggarwal Heart and Surgical Hospital Ambala City- India

**Keywords:** Viral infection, cutaneous, varicella Zoster Virus, dissemination, Erythema multiforme

## Abstract

Herpes Zoster (HZ) is a Cutaneous Viral infection caused by Varicella zoster virus (VZV). Lesions of HZ are usually limited to one dermatome only but sometimes, there can be dissemination of lesions. The present case describes the role of proper examination of HZ case, which presents with disseminated lesions.

## Introduction

Erythema multiforme (EM) is a vesiculobullous disorder with variable manifestations that predominantly affects the skin. It is regarded as a hypersensitivity disorder which is triggered by multiple factors such as infection, drugs and food [**1,2**]. The incidence of EM has been estimated to be between 0.01 and 1%. Varicella zoster associated EM is rare [**3**]. There are limited reports in literature describing the onset of EM after HZ lesion. Herpes zoster associated EM is documented to occur 10 to 14 days after the onset of Herpes Zoster (HZ) [**4**]. We report a patient who had HZ and later developed multiple EM lesions in a very short span of time. 

### Case report

A forty-four year old woman presented to us with a five days history of multiple painful papulo vesicular lesions distributed along the right D9 dermatome. She had no known co-morbidities prior to the onset of the skin lesions. She was clinically diagnosed a case of HZ. Tzanck smear performed from the lesions revealed multinucleated giant cells confirming the diagnosis. Other investigations were found within normal limits. Acyclovir therapy was deferred as she had presented on the fifth day after the onset of the lesions. She was managed symptomatically with analgesics and topical soothing agents. Lesions started healing with crusting and post inflammatory pigmentary changes.

**Fig. 1:**
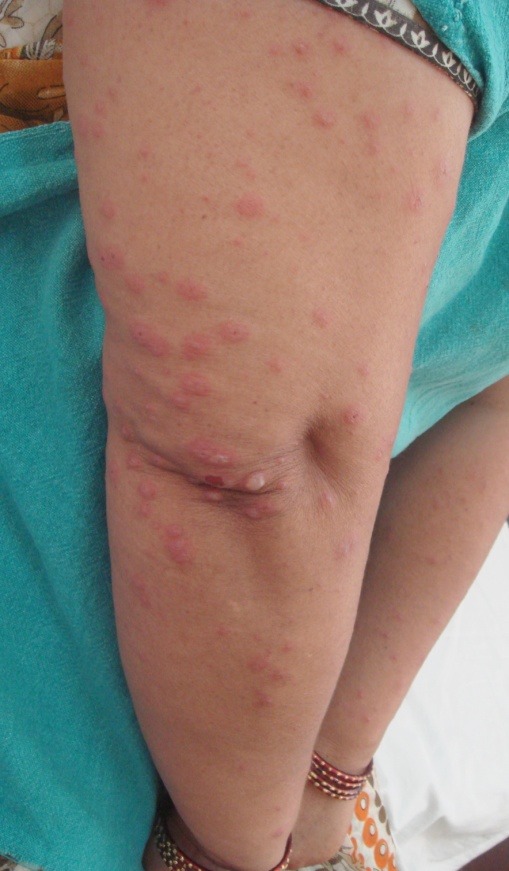
Lesions of erythema multiforme presenting as multiple erythematous papules with central hyper pigmentation and at places vesiculation

 Two days later, she reported again with multiple erythematous itchy papules all over the body. In view of dissemination of lesions, the possibility of HZ with dissemination was kept. However, on closer examination, it was observed that some of the lesions were targetoid papules and plaques with central hyper pigmentation and at places fluid filled vesicles (**Fig. 1**).

 Therefore, in view of morphology of lesions, provisional diagnosis of EM secondary to HZ was kept. She was in good health without any sign and symptom of immunosuppression. There was no mucosal involvement or lymphadenopathy. She was clinically suspected to have EM secondary to HZ. Tzanck smear prepared from new lesions did not reveal any multinucleated giant cell. Other Investigations revealed leukocytosis of 10,000/mm3 with 70% neutrophils, 1% eosinophils, and 29% lymphocytes. The C-reactive protein was of 5.9 mg/dl. Her liver function test and urine analysis were within normal limits. Her serology for Human immunodeficiency Virus, cytomegalovirus and mycoplasma were found negative. Antinuclear antibodies were also found negative. Histological examination of a skin biopsy showed a sub epidermal bulla with eosinophilic necrosis in the epidermis. A few inflammatory cells were present in the necrotic epidermis. There was a slight perivascular lymphocytic infiltrate in the papillary dermis with few histiocytes and plasma cells. There was no evidence of viral infection. These histopathological findings were consistent with our diagnosis of EM. VZV DNA studies could not be performed due to lack of availability. 

## Discussion

EM is a well-established mucocutaneous reaction to certain antigenic stimuli, usually infections or drugs. VZV is a rare cause of EM [**5**]. Two forms of VZV associated EM have been described: varicella- and zoster-associated. Varicella-associated EM occurs almost simultaneously with varicella, but the occurrence of EM after zoster is usually delayed which, usually manifests 10–14 days after the onset of HZ [**4,5**]. It has been suggested that soluble factors of T-cells may be responsible for epidermal detachment and vesicobullous eruptions in both viral lesions and EM. Identified factors involved in pathophysiology of EM-lesions are perforin, thymus- and activation-regulated chemokine (TARC), IL-12 and soluble Fas-ligand among others [**6,7**]. The present case was unique as it showed typical EM lesions due to HZ rather than with well-documented precipitating factors like herpes simplex, mycoplasma infection and drugs, including acyclovir. Moreover, our patient had onset of EM lesions very early as compared to previous reports. To the best of our knowledge, this is the first report describing the earliest onset EM in a case of HZ. The idea of reporting this case is to make the physicians aware of the fact that, the possibility of EM secondary to HZ should be kept in mind instead of only thinking of Disseminated HZ in patients of HZ with disseminated lesions.

## Conclusions

Herpes Zoster is a common entity seen in day-to-day practice. Some of the cases of HZ present with dissemination. Common causes of dissemination are Human immunodeficiency virus infection, malignancies, immunosuppressive drugs etc.

 The present case highlights the fact that the all the cases of disseminated lesions may not be of HZ, but a few may be caused by EM secondary to HZ. Therefore, we stress upon the proper examination and work up of disseminated lesions in cases of HZ.

 Funding Sources- None

IRB Approval- not needed

 Consent from patient- taken.

 Conflict of interest – The authors have no conflict of interest to disclose

 Prior presentation of paper- Not done

 Financial support/disclosure- nil 
